# Deficiency of vitamin D is associated with antenatal depression: a cross-sectional study

**DOI:** 10.47626/2237-6089-2024-0908

**Published:** 2025-09-18

**Authors:** Helena Garcia dos Santos, Aline Longoni, Jéssica Puchalski Trettim, Isabela Thurow Lemes, Júlia de Castro Menchaca, Cainá Correa do Amaral, Mariana Bonati de Matos, Luciana de Avila Quevedo, Fernanda Nedel, Gabriele Ghisleni, Diogo Onofre Souza, Ricardo Tavares Pinheiro, Adriano Martimbianco de Assis

**Affiliations:** 1 Programa de Pós-Graduação em Saúde e Comportamento Centro de Ciências da Saúde Universidade Católica de Pelotas Pelotas RS Brazil Programa de Pós-Graduação em Saúde e Comportamento, Centro de Ciências da Saúde, Universidade Católica de Pelotas (UCPel), Pelotas, RS, Brazil.; 2 Departamento de Morfologia UFPEL Pelotas RS Brazil Departamento de Morfologia, UFPEL, Pelotas, RS, Brazil.; 3 Programa de Pós-Graduação em Ciências Biológicas: Bioquímica Instituto de Ciências Básicas da Saúde Universidade Federal do Rio Grande do Sul Porto Alegre RS Brazil Programa de Pós-Graduação em Ciências Biológicas: Bioquímica, Instituto de Ciências Básicas da Saúde, Universidade Federal do Rio Grande do Sul (UFRGS), Porto Alegre, RS, Brazil.; 4 Departamento de Bioquímica UFRGS Porto Alegre RS Brazil Departamento de Bioquímica, UFRGS, Porto Alegre, RS, Brazil.

**Keywords:** Antenatal depression, pregnant women, vitamin D, major depressive episode, 25-hydroxyvitamin D

## Abstract

**Objective:**

Approximately 6 to 13% of women suffer from antenatal depression (AD) around the world. AD can lead to several health problems for mother and baby. Vitamin D is a molecule that appears to have great preventive/therapeutic potential against neuropsychiatric disorders. The present study aimed to analyze the association between vitamin D deficiency and AD in pregnant women in a city in the south of Brazil (Pelotas, Rio Grande do Sul). We hypothesize that pregnant women with a positive AD diagnosis have deficient levels of 25-hydroxyvitamin D (25(OH)D).

**Methods:**

This cross-sectional study was conducted within a cohort study (CEP/UCPEL 47807915.4.0000.5339). From this cohort, 180 pregnant women at up to 24 weeks gestation were selected (130 non-depressed and 50 depressed), and depression was diagnosed using the Mini International Neuropsychiatric Interview (MINI-Plus). Blood was collected and stored for later analysis of vitamin D 25(OH)D by the chemiluminescence method. The SPSS program was used for data analysis and results with p < 0.05 were considered statistically significant.

**Results:**

In our study, we showed a significant association between current major depressive episode in the antenatal period and vitamin D deficiency (odds ratio [OR]: 0.9; 95%CI 0.9-1.0, p = 0.003).

**Conclusion:**

Our results demonstrate that vitamin D deficiency may be involved in major depressive disorder in the antenatal period. It is thus advisable to monitor vitamin D levels during the pregnancy-puerperal cycle to minimize mental health problems in women and prevent developmental deficits in children.

## Introduction

Causing strong biological, physical, and social changes, major depressive disorder (MDD) is considered the most frequent mood disorder that occurs during pregnancy and after childbirth.^1,2^ In Brazil, the prevalence of antenatal depression (AD) varies according to the location where the study was carried out. A study conducted in the city of Pelotas using the Edinburgh Postnatal Depression Scale (EPDS) as screening instrument found that 21.1% of women had a depressive disorder during pregnancy.^3^ Another study executed in two cities in Brazil, Recife and Campinas, used the same instrument (EPDS) and found an AD prevalence of 24.3%.^4^ Depression during and after childbirth is associated with maternal factors and unfavorable fetal outcomes, including low birth weight, premature birth,^5^ and poor mother-infant interactions,^6^ and has a negative influence on the cognitive and emotional development of children.^7^

Although psychological, biological, and environmental theories of MDD are advancing, the underlying pathophysiology of AD remains unknown and it is plausible that several mechanisms are involved.^8^ Recent studies have proposed different study targets related to the pathophysiology of AD, such as the monoamine hypothesis, dysregulation of the hypothalamic-pituitary-adrenal (HPA) axis, genetic and environmental factors, increased inflammatory cytokine secretion (immunological factors), elevated levels of corticotrophin-releasing factor (CRF), and abnormalities of second messenger systems.^9-13^

Vitamin D is a molecule that appears to have great preventive/therapeutic potential for combating MDD. In a recent study in which serum vitamin D was evaluated, 85% of pregnant women and 80.5% of newborns were shown to have deficient/insufficient levels of vitamin D.^14^ Vitamin D is a steroid hormone, and its concentration in the body depends on diet and exposure to sunlight. Vitamin D can be obtained through the diet from plant sources, as ergocalciferol (D2), or from animal sources, as cholecalciferol (D3). Except for fish oil, other foods are not rich in vitamin D. Synthesis in the epidermis is one of the major sources of vitamin D and is dependent on ultraviolet (UV) radiation from sunlight.^15^ Vitamin D intake and status are low in many countries due to seasonal variations in UVB exposure.^16^

Serum 25-hydroxyvitamin D (25(OH)D) concentration is the classical marker of vitamin D status. In his literature review,^17^ Holick proposed that adults with 25(OH)D concentrations below 20 ng/mL can be considered vitamin D deficient, while 25(OH)D concentrations between 20 and 30 ng/mL in adults indicate an vitamin D insufficiency. In our study we will adopt the vitamin D deficiency/insufficiency criteria proposed by Holick.^17^ A systematic review and meta-analysis found vitamin D deficiency plays a potentially significant role in depression in the general population, indicating that low concentrations of vitamin D may be causative or predictive of depression during pregnancy and after childbirth.^18^

The present study aimed to analyze the association between serum concentration of 25(OH)D and AD in pregnant women in the urban region of Pelotas, Brazil. We hypothesized that deficiency of vitamin D during the gestational period results in a reduction of VDR activation in all cells, particularly brain cells, which induces less production of neurotransmitters and increases the likelihood of major depression episodes.

## Methods

### Design

This cross-sectional study was nested within a larger cohort study conducted in a city in southern Brazil. The central project (cohort study) began in 2016 and was carried out in multiple stages, with census tracts defined by the Instituto Brasileiro de Geografia e Estatística (IBGE) as initial sampling units. The IBGE census tracts are geographical areas that are relatively homogeneous in terms of population characteristics, economic status, living conditions, and number of inhabitants. First, the 488 census tracts from the 2010 Census (IBGE 2010) were listed for the urban area of Pelotas, a city in southern Brazil. Then, 244 of these tracts were selected by random sampling (50% of the total of the urban area). Subsequently, each of the tracts drawn were visited by the data collection team and all pregnant women living in them were identified. Women who were up to 24 weeks pregnant were invited to participate in the study. All participating pregnant women signed an informed consent form agreeing to participate in the research. More details on the sampling process can be found in publications by Pinheiro et al.^19,20^

**Figure 1:**
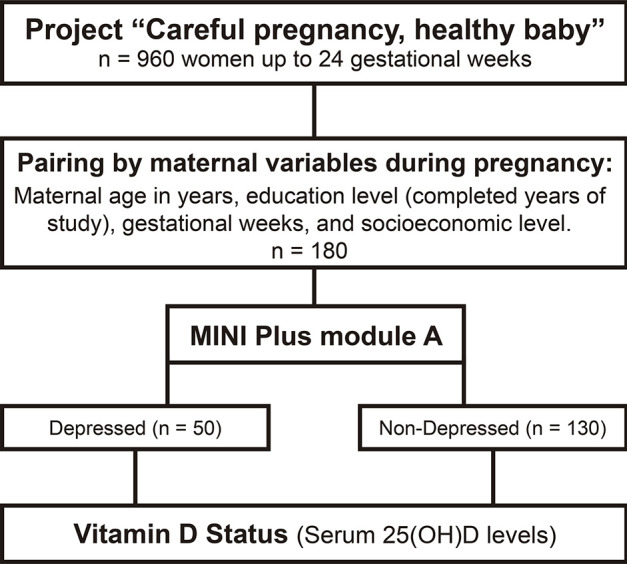
Study flowchart. Pregnant women in the urban region of Pelotas, Brazil, up to 24 gestational weeks (n = 180, depressed and non-depressed) were selected from the cohort entitled “Careful pregnancy, healthy baby” for analysis of association of serum 25-hydroxyvitamin D (25(OH)D) levels and antenatal depression (AD). The Mini International Neuropsychiatric Interview (MINI Plus Module A) was used to diagnose major depressive disorder (MDD).

First, the pregnant women were paired by maternal variables during pregnancy sourced from the central project dataset: maternal age in years, educational level (completed years of study), gestational weeks, and socioeconomic level according to the Brazilian Economic Classification Criteria proposed by the Brazilian Association of Research Companies (Associação Brasileira de Empresas de Pesquisa [ABEP]) (Figure 1). Second, we assessed the presence or absence of depression in paired pregnant women (n = 180). Third, we separated the women into two groups: non-depressed (n = 130) and depressed (n = 50). Lastly, we evaluated vitamin D status (serum 25(OH)D levels) in all pregnant women. All pregnant women identified with a current major depressive episode were referred for treatment using a brief cognitive-behavioral psychotherapy model offered by the university.^20^

### Instruments

MDD was evaluated with the “A” module of the Mini International Neuropsychiatric Interview (Mini Plus 5.0.0, Brazilian Version). Participants’ economic status was assessed according to the ABEP classification, with levels categorized as follows: A+B (high economic status), C (average economic status), and D+E (low economic status) (www.abep.org).^20^

Maternal variables, including age, ethnic group (white or other), vitamin use since becoming pregnant (yes/no), and gestational age in weeks were collected via a structured general questionnaire.

The nutritional status of the pregnant women was assessed according to Atalah et al.,^21^ taking into account gestational age and the pregnant woman’s current body mass index (BMI), showing, based on this information, the ideal weight gain for the period. The classification is given by the BMI X gestational age curve, classifying nutritional status into low weight, normal weight, overweight, or obesity. For our study, the continuous BMI score was used.^22^ The BMI was evaluated by weight/height^2^ (kg/m^2^), with weight measured with an anthropometric scale and height measured with a stadiometer.

### Blood sample collection and processing

Blood samples were obtained from all pregnant women (n = 180) by venipuncture (10 mL). The blood sample was immediately centrifuged at 3,000 × *g* for 10 min, and the supernatant was transferred to tubes and stored at -80 °C for further analysis of vitamin D 25(OH)D at a clinical analysis laboratory. Participants were asked to fast for 8-10 hours before blood collection, and 20 mL of peripheral venous blood was collected from each participant.^23^

### Measurement of serum 25(OH)D by chemiluminescence

Serum 25(OH)D concentrations (ng/mL) were measured using the microparticle chemiluminescence method according to the manufacturer’s instructions using Atellica^®^ IM (Siemens, Erlangen, BV, Germany). Atellica^®^ IM is an accurate and precise assay with a functional sensitivity of ≤ 3.0 ng/mL and inter-assay imprecision of ≤ 20%.^24^ It has been certified since 2018 for total 25(OH)D assays by the Centers for Disease Control and Prevention (CDC) Vitamin D Standardization Certification Program.^25^ Serum 25(OH)D concentrations were classified by cut-off values into sufficient (> 30 ng/mL), insufficient (20-30 ng/mL), and deficient (< 20 ng/mL).^17^

### Statistical analyses

Data on independent variables were collected with questionnaires and coded and double-entered in EpiData 3.1 to check for inconsistencies. To describe the characteristics of the sample, simple and relative frequencies were used for the categorical variables and means and standard deviations (SD) were used to describe the outcomes. Covariant analysis was performed using Student’s *t* test and analysis of variance (ANOVA), including variables with p < 0.20 in the crude analysis in the multivariate analysis by logistic regression (backward method), with results presented as odds ratio (OR) and 95% confidence intervals (95%CIs). Associations with p < 0.05 were considered statistically significant.

### Ethical considerations

The project to which this study is linked was approved by the Universidade Católica de Pelotas Research Ethics Committee under protocol number 47807915.4.0000.5339, process number 1.729.653.

## Results

Table 1 presents the sample characteristics and the covariant analysis results. The mean vitamin D level among the 180 pregnant women analyzed was 20.1 ± 6.1 ng/mL. Regarding the characteristics of the sample assessed by univariate analysis, most pregnant women were between 24 and 29 years old (n = 77, 42.8%), more than half (n = 110, 61.1%) were overweight or obese, the vast majority had white ethnicity (n = 114, 73.5%), were in the second trimester (71.1% of the total sample), and belonged to economic class C (n = 119, 67.2%). Regarding current major depressive episode, 50 (27.8%) participants were diagnosed with depression. Concerning use of vitamins since becoming pregnant, only 53 (29.4%) women reported taking vitamins. Additionally, as shown in Table 1, the covariant analysis (ANOVA) showed statistical differences (p < 0.05) between those with and without current major depressive episode for serum vitamin D concentration, ethnic group, and psychological/psychiatric treatment. The other variables, including age, nutritional status, economic class, gestational trimester, and use of vitamins since becoming pregnant, were not different in relation to the outcome (p > 0.05).

**Table 1:** Sample characteristics and covariant analysis of pregnant women in the urban region of Pelotas, Brazil

Variable	Total n = 180	Depressed n = 50	Non-depressed n = 130	p-value*
Age, years				0.478
Up to 23	41 (22.8)	10 (20.0)	31 (23.8)	
From 24 to 29	77 (42.8)	25 (50.0)	52 (40.0)	
30 or older	62 (34.4)	15 (30.0)	47 (36.2)	
				
Ethnic group^†^				0.045
White	114 (73.5)	26 (61.9)	88 (79.9)	
Non-white	41 (26.5)	16 (38.1)	25 (22.1)	
				
Nutritional status				0.965
Low weight	16 (8.9)	04 (8.0)	12 (9.2)	
Healthy weight	54 (30.0)	15 (30.0)	39 (30.0)	
Overweight/obesity	110 (61.1)	31 (62.0)	79 (60.8)	
				
Economic class^†^				0.933
High (A/B)	35 (19.8)	10 (20.4)	25 (19.5)	
Middle (C)	119 (67.2)	32 (65.3)	87 (68.0)	
Low (D/E)	23 (13.0)	07 (14.3)	16 (12.5)	
				
Gestational trimester				0.295
1st	51 (28.3)	17 (34.0)	34 (26.2)	
2nd	129 (71.7)	33 (66.0)	96 (73.8)	
				
Major depressive episode in the past				0.012
No	166 (92.2)	50 (100.0)	116 (89.2)	
Yes	14 (7.8)	00 (0.0)	14 (10.8)	
				
Psychological/psychiatric treatment				0.066
No	176 (97.8)	47 (94.0)	129 (99.2)	
Yes	04 (2.2)	03 (6.0)	01 (0.8)	
				
Vitamin use since becoming pregnant				0.174
No	127 (70.6)	39 (78.0)	88 (67.7)	
Yes	53 (29.4)	11 (22.0)	42 (32.3)	
				
Serum vitamin D concentration, mean (SD)	20.1 (6.1)^‡^	17.7 (5.2)^‡^	21.1 (6.1)	0.001

Data presented as n (%).

SD = standard deviation.

* Student’s *t* test and analysis of variance (ANOVA).

^†^ Variables with missing data; ^‡^ ng/mL.

Table 2 shows the analysis, adjusted by linear regression, of variables with p < 0.20 in the covariant analysis: serum vitamin D concentration, ethnic group, and psychological/psychiatric treatment. Of these, only serum vitamin D is associated with current major depressive episode (OR: 0.9; 95%CI 0.9-1.0, p = 0.003).

**Table 2:** Serum vitamin D concentration are associated with the outcome (current) major depressive episode in the multivariate analysis by logistic regression (95%CIs) of independent variables with p < 0.20 among pregnant women from the urban region of Pelotas, Brazil

Variable	OR	95%CI	p-value
Serum vitamin D concentration	0.9	0.9-1.0	0.003*
Ethnic group (White^†^)	2.0	0.9-4.3	0.100
Psychological/psychiatric treatment (no^†^)	4.1	0.3-51.9	0.270

95%CI = 95% confidence interval; OR = odds ratio.

* Associations with p < 0.05 were considered statistically significant.

^†^ Reference category.

**Figure 2:**
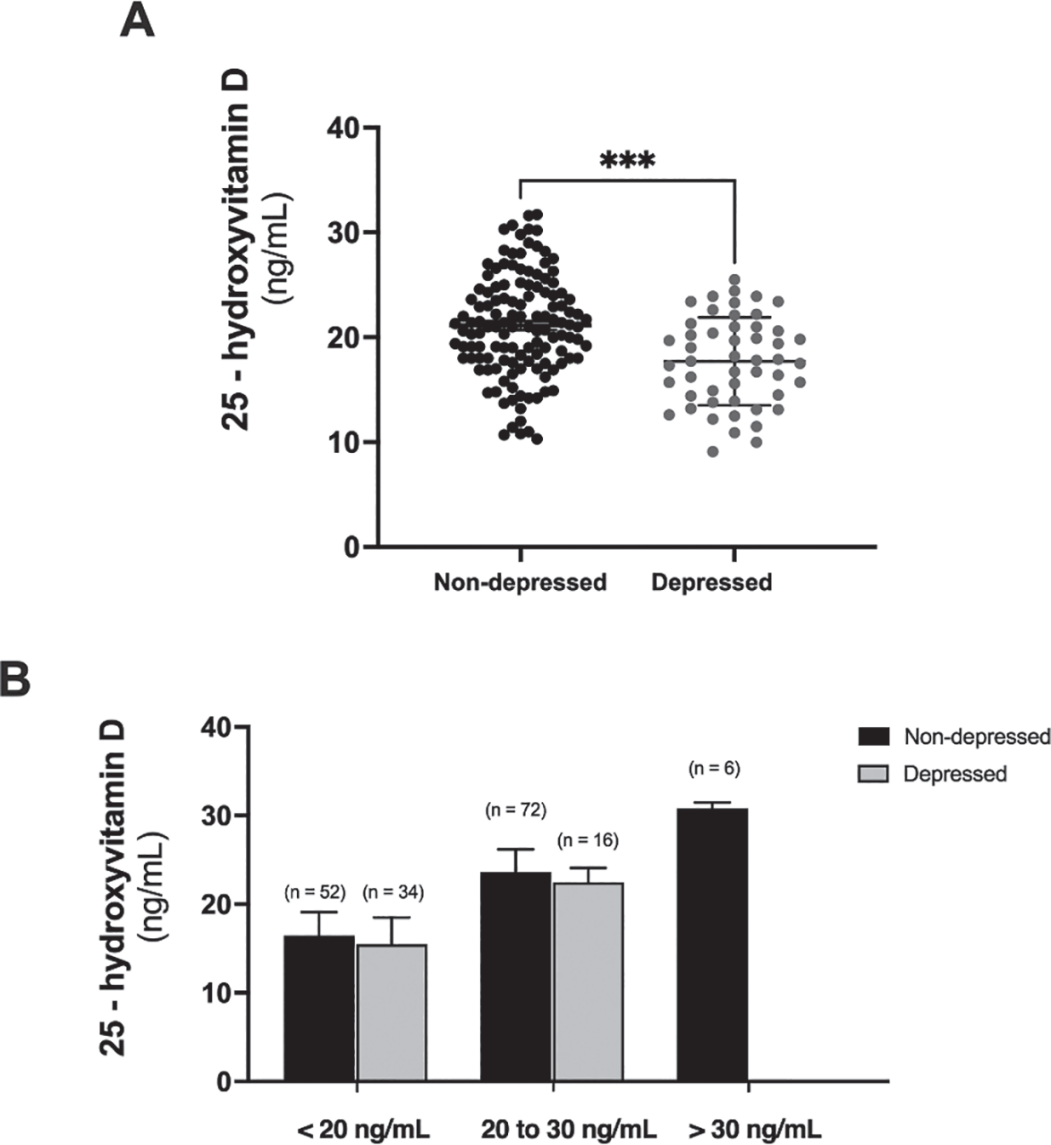
Depressed pregnant women in the urban region of Pelotas, Brazil, showed vitamin D deficiency. (A) Covariant analysis by Student’s *t* test of the serum 25-hydroxyvitamin D (25(OH)D) levels in pregnant women (depressed, n = 50 and non-depressed, n = 130). *** p < 0.001. (B) Prevalence of depressed and non-depressed pregnant women allocated by serum 25(OH)D cut-off values to sufficient (>30 ng/mL), insufficient (20-30 ng/mL), or deficient (< 20 ng/mL).

As illustrated in Figure 2A, it was found that depressed pregnant women had significantly lower serum vitamin D levels compared to non-depressed pregnant women. The non-depressed group had an average serum vitamin D level of 21.1 ± 6.1 ng/mL, whereas the depressed group had an average of 17.7 ± 5.2 ng/mL. This difference was statistically significant (p =0.001). In Figure 2B, non-depressed and depressed pregnant women were categorized according to the serum 25(OH)D cut-off values proposed by Holick.^17^ Although no statistical difference was found in this comparison, it was observed that the majority of depressed pregnant women (n = 34, 68%) had 25(OH)D levels lower than 20 ng/mL. Conversely, the majority of non-depressed pregnant women (n = 72, 55.4%) had serum 25(OH)D levels ranging from 20 to 30 ng/mL.

In our study, the sample of pregnant women was in the interval from gestational weeks 5 to 24. Supplementary Table S1 contains the vitamin D concentration per gestational week of the pregnant women included in the study. Statistical analysis demonstrated no difference in vitamin D concentration by gestational week.

## Discussion

Pregnancy is a life event accompanied by numerous psychological and physiological changes that increase vulnerability to the onset or recurrence of mental disorders, with AD being one of the most prevalent psychiatric disorders with adverse impacts on the health of both mother and baby.^26^ Vitamin D deficiency is one of the most prevalent micronutrient deficiencies among pregnant women. In our cross-sectional study, we confirmed the working hypothesis, since pregnant women with AD had vitamin D deficiency. In addition, we observed that increasing age of pregnant women was significantly related to higher vitamin D levels. Together, our results have clinical importance, since vitamin D is associated with numerous important physiological functions, especially during the pregnancy-puerperal cycle, and its deficiency is directly associated with neuropsychiatric disorders such as AD.

High rates of insufficient/deficient serum vitamin D have been reported in several countries around the world. Some studies report that 88% of the population has serum vitamin D lower than 30 ng/mL, 37% have vitamin D lower than 20 ng/mL, and up to 7% have vitamin D under 10 ng/mL.^27,28^ Various societies in several countries determine different cut-offs for definition of deficient, insufficient, and sufficient vitamin D. In our work, we considered the following deficient, insufficient, and sufficient serum vitamin D levels: below 20 ng/mL as deficient, between 20 to 30 ng/mL as insufficient, and above 30 to 50 ng/mL as sufficient for health benefits.^29,30^ Our study demonstrated that depressed pregnant women had lower vitamin D levels compared to non-depressed pregnant women (depressed: 21.1 ± 6.1 ng/mL vs. non-depressed: 17.7 ± 5.2 ng/mL) (Figure 2). When we plot the vitamin D level results according to the 25(OH)D cut-off values proposed by Holick,^17^ (Figure 2B) we can observe that the majority of depressed pregnant women have 25(OH)D levels below 20 ng/mL (depressed, 15.49 ± 2.99 ng/mL: 68%), which represents deficient levels of this vitamin. However, we can see that most non-depressed pregnant women have 25(OH)D levels between 20 and 30 ng/mL (23.63 ± 2.66 ng/mL: 55,4%), representing insufficient levels of vitamin D. Our data demonstrate that in addition to an association between AD and decreased levels of vitamin D, most depressed pregnant women have deficient levels of vitamin D. We believe that these results are linked, in part, to the fact that Pelotas is located in the extreme southern region of Brazil and therefore has a more severe and longer-lasting winter and the population of this region therefore has less exposure to the sun.

Studies indicate that pregnant women are more likely to have low concentrations of vitamin D when compared to nonpregnant women^31^ and that 25(OH)D can vary between trimesters, with lower levels in the 1st and 3rd trimesters than in the second trimester.^32^ Physiological vitamin D metabolism during pregnancy differs from that of a non-pregnant woman.^33^ Heaney et al.^34^ showed that conversion of vitamin D to 25(OH)D was unchanged during pregnancy, therefore, the conversion of 25(OH)D to 1.25(OH)_2_D is significantly altered in this period. This change that occurs in the metabolism of vitamin D (conversion of 25(OH)D to 1.25(OH)_2_D) is unique and is not seen at any other stage of life. Hollis et al.^35^ demonstrated that the serum concentration of 1.25(OH)2D at 12 weeks of gestation is increased more than twice compared to a non-pregnant woman and this increase continues in the following gestational weeks. In Supplementary Table S1 we compare 25(OH)D concentrations by gestational week of all the women included in the study. We can observe that, despite there being fluctuations in vitamin D concentrations throughout the gestational weeks, there was no significant difference.

A recent study that examined the association between vitamin D and perinatal depression in Chinese pregnant and lactating women suggested a significant association between vitamin D status and *postpartum depression*; however, the association between vitamin D status and AD was not significant.^36^ In another study, the researchers found a potential role of vitamin D deficiency in depression in the general population, indicating that low vitamin D may be causative or predictive of depression during pregnancy and after childbirth.^18^ In a recent systematic review that analyzed seven studies measuring vitamin D during pregnancy or 24 hours after delivery, six studies showed conclusive results indicating that lower 25(OH)D levels were related to *postpartum depression*.^37^ On the other hand, several randomized clinical trials have been carried out to propose a safe dosage of vitamin D supplementation to minimize the effects of vitamin D deficiency. These studies demonstrated that a 4,000 IU dose of vitamin D3/day safely elevates circulating 25(OH)D to a level that, regardless of race, fully restores vitamin D metabolism in pregnant women.^35,38,39^

Numerous biological mechanisms may explain the association observed between 25(OH)D concentrations and AD. Depression is classically associated with dysregulated HPA axis function, increased inflammatory markers, oxidative stress, and overactivity of the sympathoadrenal system.^40^ Vitamin D may beneficially act on three proposed pathways responsible for the development of perinatal depression: 1) by reducing the production of pro-inflammatory cytokines via inhibition of the *NF-κB* gene through the binding of vitamin D with its nuclear receptor (nVDR); 2) by inhibiting the release of corticotropin-releasing hormone (CRH) from the hypothalamus, thus decreasing secretion of adrenocorticotropic hormone (ACTH) by the anterior pituitary gland, resulting in reduced synthesis of cortisol by the adrenal gland; and 3) by increasing calcium metabolism, which stimulates secretion of gonadotropin-releasing hormone (GnRH) by the hypothalamus. GnRH stimulates the release of follicle-stimulating hormone (FSH) and luteinizing hormone (LH) by the pituitary gland, which act on the gonads by stimulating production of estriol.^41-43^

Our study had some limitations, including the small sample size, the lack of information about previous use of vitamin D or calcium, diet composition, skin tone, or the amount of sun exposure that could alter vitamin D levels. However, our results have translational importance, as we demonstrate a significant association between vitamin D deficiency and AD in pregnant women (Figure 2). Current major depressive episode during pregnancy results in several health risks for the pregnant woman and her child after birth.^44^ Based on our work, we propose that new studies evaluate pregnant women for longer periods in longitudinal studies or perform vitamin D supplementation in randomized clinical trials. Moreover, guidelines and public policy protocols should be created to control vitamin D status during pregnancy in developing countries.

## Supplementary Material

Supplementary Material
