# Supplementary material for: Deficiency of vitamin D is associated with antenatal depression: a cross-sectional study
Source: Trends Psychiatry Psychother. 2025 Sep 18;47:e20240908. doi: 10.47626/2237-6089-2024-0908 (PMC12962367; doi:10.47626/2237-6089-2024-0908)
Supplement: Supplementary Material [file 2238-0019-trends-47-e20240908-suppl01.pdf]

http://doi.org/10.47626/2237-6089-2024-0908

Supplementary Table S1 - Analysis of vitamin D per gestational week of pregnant women in the urban region of Pelotas, Brazil.

Gestational week	Vitamin D Mean (ng/ml)	N	Standard deviation
5	26.00	1	-
6	22.82	5	9.87
7	21.53	7	4.37
8	20.36	5	7.76
9	19.80	10	4.19
10	17.76	6	6.12
11	22.17	7	4.63
12	17.07	10	5.35
13	18.38	13	5.04
14	18.26	5	5.62
15	19.33	6	6.99
16	20.76	15	5.08
17	24.28	9	8.63
18	20.31	12	6.45
19	23.05	7	5.40
20	20.90	14	6.53
21	21.16	12	6.97
22	18.92	11	4.55
23	21.50	11	5.86
24	16.52	14	5.28
**Total**	**20.15**	**180**	**6.03**

Analysis of variance (ANOVA): p = 0.336.
